# Association of *a priori* dietary patterns with depressive symptoms: a harmonised meta-analysis of observational studies

**DOI:** 10.1017/S0033291719001958

**Published:** 2020-08

**Authors:** Mary Nicolaou, Marco Colpo, Esther Vermeulen, Liset E. M. Elstgeest, Mieke Cabout, Deborah Gibson-Smith, Anika Knuppel, Giovana Sini, Danielle A. J. M. Schoenaker, Gita D. Mishra, Anja Lok, Brenda W. J. H. Penninx, Stefania Bandinelli, Eric J. Brunner, Aiko H. Zwinderman, Ingeborg A. Brouwer, Marjolein Visser

**Affiliations:** 1 Department of Public Health, Amsterdam UMC, University of Amsterdam, Amsterdam Public Health Research Institute, Meibergdreef 9, Amsterdam, the Netherlands; 2 Azienda USL Toscana Centro, InCHIANTI Study Group, Florence, Italy; 3 Department of Health Sciences, Faculty of Science, Vrije Universiteit, Amsterdam Public Health Research Institute, Amsterdam, the Netherlands; 4 Department of Psychiatry, Amsterdam UMC, Vrije Universiteit Amsterdam, Amsterdam Public Health Research Institute, de Boelelaan 1117, Amsterdam, the Netherlands; 5 Research Department of Epidemiology and Public Health, University College London, UK; 6 School of Public Health, Faculty of Medicine, University of Queensland, Brisbane, Queensland, Australia; 7 Centre for Behavioral Research in Cancer, Cancer Council Victoria, Melbourne, Victoria, Australia; 8 Department of Psychiatry, Amsterdam UMC, University of Amsterdam, Amsterdam Public Health Research Institute, Meibergdreef 9, Amsterdam, the Netherlands; 9 Department of Clinical Epidemiology, Biostatistics and Bioinformatics, Amsterdam UMC, University of Amsterdam, Meibergdreef 9, Amsterdam, the Netherlands

**Keywords:** AHEI-2010, DASH, depression, diet, Mediterranean diet, meta-analysis, MooDFOOD project

## Abstract

**Background:**

Review findings on the role of dietary patterns in preventing depression are inconsistent, possibly due to variation in assessment of dietary exposure and depression. We studied the association between dietary patterns and depressive symptoms in six population-based cohorts and meta-analysed the findings using a standardised approach that defined dietary exposure, depression assessment and covariates.

**Methods:**

Included were cross-sectional data from 23 026 participants in six cohorts: InCHIANTI (Italy), LASA, NESDA, HELIUS (the Netherlands), ALSWH (Australia) and Whitehall II (UK). Analysis of incidence was based on three cohorts with repeated measures of depressive symptoms at 5–6 years of follow-up in 10 721 participants: Whitehall II, InCHIANTI, ALSWH. Three *a priori* dietary patterns, Mediterranean diet score (MDS), Alternative Healthy Eating Index (AHEI-2010), and the Dietary Approaches to Stop Hypertension (DASH) diet were investigated in relation to depressive symptoms. Analyses at the cohort-level adjusted for a fixed set of confounders, meta-analysis used a random-effects model.

**Results:**

Cross-sectional and prospective analyses showed statistically significant inverse associations of the three dietary patterns with depressive symptoms (continuous and dichotomous). In cross-sectional analysis, the association of diet with depressive symptoms using a cut-off yielded an adjusted OR of 0.87 (95% confidence interval 0.84–0.91) for MDS, 0.93 (0.88–0.98) for AHEI-2010, and 0.94 (0.87–1.01) for DASH. Similar associations were observed prospectively: 0.88 (0.80–0.96) for MDS; 0.95 (0.84–1.06) for AHEI-2010; 0.90 (0.84–0.97) for DASH.

**Conclusion:**

Population-scale observational evidence indicates that adults following a healthy dietary pattern have fewer depressive symptoms and lower risk of developing depressive symptoms.

## Introduction

Depression is a severe mental disorder predicted to be the second leading cause of disability globally by 2020 (Global Burden of Disease study 2013 collaborators, 2015). Like other mental disorders, it has multi-causal pathogenesis including genetic, environmental and lifestyle factors.

Evidence has accumulated to suggest that diet plays a role in the onset of depression, resulting in increased interest in the potential of dietary approaches for the prevention and treatment of depression (Sarris *et al*., 2015; Molendijk *et al*., 2018). Hypothesised mechanisms for the action of diet include direct influence of nutrients on brain metabolism, inflammation and oxidative stress (Lopresti *et al*., 2013). Several nutrients including essential fatty acids (eicosapentaenoic and docosahexaenoic acid), vitamins B_12_, B_6_, folate and vitamin D, magnesium, selenium and zinc (Almeida *et al*., 2015; Mocking *et al*., 2016; Appleton *et al*., 2017; Schefft *et al*., 2017) have been investigated in relation to depression; however, the findings are mixed (Schefft *et al*., 2017). The challenge of separating the different nutrient-health associations in observational studies is acknowledged. Thus dietary patterns may be more informative for investigating diet–disease relationships as they capture the synergistic and correlated effects of separate nutrients and foods (Ocké, 2013).

Existing systematic reviews of surveys and cohort studies provide inconclusive evidence that healthy dietary patterns, consisting of high intakes of vegetables, fruit, fish, healthy oils and low intakes of red and processed meat are protective for development of depressive symptoms (Quirk *et al*., 2013; Lai *et al*., 2014; Rahe *et al*., 2014; Khalid *et al*., 2016; Lassale *et al*., 2018; Molendijk *et al*., 2018). The conflicting results to date may be due to differences in study design, including choice of instrument utilised to measure depressive symptoms and approach to adjustment for potential confounders. Further, the identification of food groups is unstandardised across studies, e.g. low and high-fat dairy intakes may be combined, and dietary patterns may be operationalised in different ways, using *a priori* or *a posteriori* methods. Such methodological issues have implications for the apparent effects of ‘healthy diets’, as variously defined. Three previous meta-analyses of observational studies (Quirk *et al*., 2013; Lassale *et al*., 2018; Molendijk *et al*., 2018) found inconsistencies in the associations observed, with moderate to high levels of heterogeneity.

We aimed to clarify the potential of healthy diets in the prevention of depression using data from observational studies. We examined cross-sectional and prospective associations between three *a priori* healthy dietary patterns and depressive symptoms in six cohorts using a standardised protocol to derive summary statistics for meta-analysis.

## Methods

We included five cohort studies that were part of the MooDFOOD project consortium: the Invecchiare in Chianti study (InCHIANTI); the Longitudinal Aging Study Amsterdam (LASA); Netherlands Study of Depression and Anxiety (NESDA); Healthy Life in an Urban Setting (HELIUS); the Whitehall II study; and an external cohort, the Australian Longitudinal Study on Women's Health (ALSWH).

InCHIANTI is an ongoing population-based study performed in Italy. Baseline data collection started in 1998 and recruited 1453 participants aged 20–102 years. Follow-up data were collected every 3 years until 2012 (Ferrucci *et al*., 2000). LASA includes a nationally representative sample of older adults (⩾55 years) in the Netherlands. Baseline data collection of the first cohort (*N* = 3107) started in 1992 and additional cohorts aged 55–65 years were added in 2002 (*N* = 1002) and 2012 (*N* = 1023) (Hoogendijk *et al*., 2016). NESDA is a naturalistic cohort representative of those with various stages of depressive and anxiety disorders, sourced from different health care settings in the Netherlands. It included 2981 participants aged 18–65 years at baseline (2008) and follow-up is ongoing (Penninx *et al*., 2008). HELIUS is a study of health among an urban multi-ethnic population in Amsterdam, the Netherlands. Baseline data collection started in 2011 and included 24 789 persons aged 18–70 years with Surinamese, Turkish, Ghanaian, Moroccan and Dutch ethnicity. Follow-up is ongoing (Snijder *et al*., 2017). The Whitehall II study, with baseline in 1985–88, recruited 10 308 British London-based civil servants aged 35–55 years. Follow-up data was collected every 2 years by questionnaire, with 5-yearly research clinics (including measurement of diet) (Marmot *et al*., 1991). ALSWH is an ongoing national cohort study in Australia. We used data from the 13 714 women in the 1946–41 cohort who were aged 45–50 years when recruited in 1996. Follow up is ongoing with questionnaires every 2–3 years (Lee *et al*., 2005).

### Study protocol

We developed a standardised protocol to define the analytic sample, create the determinant and outcome variables, select confounders and perform statistical analyses. The protocol was shared with consortium members and approved prior to starting the analyses. Three studies measured diet and depressive symptoms at a single moment (LASA, NESDA, HELIUS), while the other three studies had measures of depression at many moments. In prospective analyses we used data from Whitehall II at phase 7 (2002–2004) and phase 9 (2007–2009), InCHIANTI: baseline (1998–2000) and phase 2 (2004–2006), and ALSWH: survey 3 (2001) and survey 5 (2007). The choice of cohort phase to be included in this analysis was based on whether diet and depressive symptoms were included in what was considered to be the ‘baseline’; similar follow-up time; and if possible, comparable age range of participants.

### Measures of depressive symptoms

Four cohorts measured depressive symptoms using the Centre for Epidemiologic Studies Depression (CESD) scale: Whitehall II, LASA and InCHIANTI used CESD-20 and ALSWH used CESD-10 (Radloff, 1977). In NESDA depressive symptoms were assessed with the 30-item Inventory of Depressive Symptomatology-Self Report (IDS) (Rush *et al*., 1996). HELIUS measured depressive symptoms with the Patient Health Questionnaire (PHQ-9) (Kroenke *et al*., 2001). Anti-depressive medication was self-reported, except in HELIUS where research staff noted medication as brought in by participants. Definitions used in the current study:

1. ‘Depressive symptoms’ – continuous variable based on the number of depressive symptoms.
2. ‘High depressive symptoms’ – dichotomous variable based on validated cohort-specific cut-offs. CES-D cut-off of ⩾20 was applied by InCHIANTI (Fava, 1983); CES-D ⩾16 was used by Whitehall II and LASA (Radloff, 1977; Beekman *et al*., 1997); ALSWH ⩾10 for the CESD-10 (Andresen *et al*., 1994). NESDA defined high depressive symptoms as IDS ⩾14 (Gibson-Smith *et al*., 2018), HELIUS as PHQ-9 ⩾10 (Martin *et al*., 2006; Galenkamp *et al*., 2018).
3. ‘High depressive symptoms e/o meds’ – high depressive symptoms and/or use of anti-depressive medication.

### Measurement of diet and definition of dietary patterns

HELIUS, LASA and NESDA used the HELIUS 238-item, FFQ (Beukers *et al*., 2015). Whitehall II (127-item) and ALWSH (121-item) used FFQs from the Nurse's Health Study (Willett *et al*., 1985) adapted for their national context (Ireland *et al*., 1994; Brunner *et al*., 2001) and InCHIANTI used a country-specific 248-item FFQ from the European Prospective Investigation into Cancer and Nutrition study (Pisani *et al*., 1997). Grouping of foods was based on the protocol developed for this study. See online Supplementary file 1, Table S1.

We selected three *a priori* defined dietary patterns that are commonly used in studies of diet and depression:

• Mediterranean Diet Score (MDS) based on Panagiotakos *et al*. (2007).
• Alternative Healthy Eating Index 2010 (AHEI-2010) based on Chiuve *et al*. (2012).
• Dietary Approaches to Stop Hypertension (DASH) based on Fung *et al*. (2008).

See online Supplementary file 1, Table S2 for a description of dietary patterns.

### Exclusions

In the cohorts including older persons (LASA, Whitehall II and InCHIANTI) we excluded participants with cognitive impairment based on the Mini Mental State Examination score <24 as this may have caused an unreliable dietary intake assessment (Tombaugh and McIntyre, 1992). We also excluded participants with improbable energy intakes based on Willett's cut-offs (women: <500 kcal, >3500 kcal and men: <800 kcal, >4000 kcal) (Willett, 2013).

### Confounders

Confounders were selected based on the literature and included age, sex and ethnicity (measured in HELIUS and Whitehall II), education level (highest completed educational level in three categories, based on cohort-specific measures), marital status (married/living with partner, never married, divorced/widowed), employment situation [in paid employment, not working (home-maker, retired), unemployed or unable to work (sickness benefit)], smoking (current, former, never), physical activity (three categories based on cohort-specific measures), energy intake, self-reported chronic disease as defined by the individual cohorts (type-2 diabetes, cancer, cardiovascular disease), body mass index (BMI, kg/m^2^) based on self-reported or measured body height and body weight, and waist circumference (WC, cm).

### Analysis at the individual study level

All variables (confounder, dietary and depression variables), including categorical variables, were standardised for the statistical analysis. Models were built stepwise to allow evaluation of the biasing effect of confounders. Model zero included age, sex, ethnicity and educational level. Model one added marital status and employment situation. Model two added lifestyle factors: smoking, physical activity, energy intake. Model three additionally accounted for chronic disease. Finally, model four added BMI, online Supplement 2 presents the results of all analyses.

#### Cross-sectional analysis

The association of dietary patterns with the transformed, standardised continuous ‘depressive symptoms’ *score* was modelled using linear regression. We chose to transform the depressive symptoms score for analysis as its association with the dietary patterns did not produce normally distributed residuals. The association of dietary pattern and the *dichotomous variables* ‘high depressive symptoms’ and ‘high depressive symptoms e/o meds’ was analysed using logistic regression.

Prospective analysis followed the same approach and considered the association of dietary pattern at baseline with depressive symptoms at follow-up. In regression analysis, we corrected for continuous depressive symptoms score at baseline, in logistic regression we excluded those with high depressive symptoms at baseline. We tested for interaction with age, sex (in all studies) and ethnicity (in the Whitehall II and HELIUS studies only as the other cohorts did not include sufficient numbers of different ethnic groups to allow these analyses). There was no consistent pattern of interaction, so final analyses do not include interaction terms. To illustrate, in LASA there was statistically significant interaction with sex in the association between AHEI-2010 and continuous depressive symptoms, in Whitehall interaction with sex was observed in for the MDS only, while in other cohorts we observed no interactions with sex.

#### Sensitivity analyses

The continuous ‘depressive symptoms score’ was not normally distributed in most cases so we transformed the standardised depression score (using an LN + 1 transformation). However, the transformation applied was not successful in normalising the depression scores in all cohorts so in sensitivity analyses we studied the diet–depression relationship using partial Spearman's correlations. We also ran the analyses with the addition of WC instead of BMI to model four as this measure is sometimes considered a better proxy for body fat levels.

### Meta-analysis

A random-effects approach was considered to be the best option to analyse regression coefficients and ORs because data were derived from observational studies with different recruitment and examination protocols and population characteristics. lnORs were obtained by the logistic regression coefficients and standard errors approximated by delta-method (Casella and Berger, 2002). The inverse-variance method was applied to give preferences to large samples and Restricted Maximum Likelihood Method was applied to estimate the between-study variance (Peace and Chen, 2013). Heterogeneity between studies was quantified by *I*^2^ statistic and tested by Cochran's *Q* test (Higgings and Thompson, 2002). Analyses were performed in R (R Development Core Team, 2013) using the Metafor package (Viechtbauer, 2010).

## Results

Participant characteristics are presented in Table 1. LASA and InCHIANTI participants were generally the oldest (mean ages 69.3 and 65.1 respectively). Distribution of sex differed between cohorts, ALSWH included only women, whereas Whitehall II predominantly included men. Other characteristics reflect differences in age, sex and ethnic composition of the different populations. The prevalence of ‘high depressive symptoms’ ranged from 13% in HELIUS to 20.9% in ALSWH with the highest prevalence in NESDA (43%), consistent with the nature of this latter study population.

**Table 1.** Population characteristics

	InCHIANTI *n* = 1050	LASA *n* = 1269	Whitehall II *n* = 4633	NESDA *n* = 1618	HELIUS *n* = 4619	ALSWH *n* = 9918
Age (years), mean (s.d.)	65.3 (15.6)	69.3 (8.35)	61.2 (5.97)	52.0 (13.2)	46.4 (12.6)	52.5 (1.5)
Women %	51.6	51.6	26.5	67.7	59.6	100.0
Educational level %						
Low	76.8	11.8	18.2	3.8	35.2	64.1
Middle	19.0	58.2	28.9	46.5	28.5	20.5
High	4.3	30.0	53.0	49.6	36.3	15.4
Marital status %						
Married/cohabitating	67.0	72.4	77.1	50.3	53.8	81.8
Single/never married	12.4	7.5	12.1	31.6	30.4	3.0
Divorced/widowed	20.6	20.1	10.7	18.0	15.9	15.2
Employment %						
Employed	20.9	37.3	51.2	57.4	65.2	64.1
Not part of labour force or retired	70.1	57.1	47.0	22.1	16.7	27.8
Unemployed/disability	9.0	5.7	1.8	20.5	18.1	8.1
Smoking status %						
Current smoker	21.8	12.0	48.5	23.1	21.7	14.4
Former smoker	26.2	59.7	44.5	43.2	25.9	24.5
Never	52	28.3	7.01	33.7	52.4	61.1
Physical activity %^a^						
Low	12.1	33.3	11.3	33.3	33.4	33.3
Medium	78.1	33.3	56.5	33.3	33.3	33.4
High	9.8	33.3	32.3	33.4	33.3	33.3
Self-reported CVD %	59.1	24.4	9.8	10.5	11.6	4.7
Self-reported T2DM %	9.4	8.5	4.2	6.0	10.7	4.8
Self-reported cancer %	4.6	14.3	5.5	7.8	1.9	22.2
WC (cm) mean (s.d.)	91.4 (10.7)	97.7 (11.7)	91.2 (12.1)	93.1 (14.3)	91.7 (12.9)	N.A.
BMI (kg/m^2^) mean (s.d.)	27.1 (3.9)	27.2 (4.2)	26.6 (4.2)	26.3 (4.8)	26.7 (5.0)	26.8 (5.5)
Energy intake (kcal/d) Mean (s.d.)	2043 (577)	2085 (571)	2154 (586)	2135 (601)	2089 (682)	1578 (520)
Depressive symptoms^b^ sum (median, IQR)	9 (11)	8.0 (9.0)	6.0 (9.0)	11.0 (15)	3.0 (5.0)	4.6 (6.1)
High depressive symptoms, *n* (%) at baseline	173 (16.5)	199 (15.7)	615 (13.3)	689 (43.0)	599 (13.0)	2073 (20.9)
High depressive symptoms and/or meds at baseline, *n* (%)	193 (18.4)	239 (18.8)	701 (15.1)	762 (52.4)	707 (15.3)	2380 (24.0)
High depressive symptoms at follow-up, *n* (%)	73 (10.5)	n/a	447 (10.7)	n/a	n/a	2446 (28.5)

a Physical activity variable was tertiles of MET minutes per week in LASA, NESDA, HELIUS and ALSWH. InCHIANTI and Whitehall II used study specific cut-offs to define the three different levels.

b Depressive symptoms measured using the CESD-20 InCHIANTI, LASA, Whitehall II, CESD-10 in ALSWH, IDS in NESDA, PHQ-9 in HELIUS.

Table 2 presents an overview of the dietary pattern scores. Direct comparison of the cohorts is not possible due to differences in the variables measured by different studies. Specifically, sodium intake was not measured in LASA, NESDA and HELIUS (included in the DASH and the AHEI-2010 scores), trans-fat intake was not calculated in InCHIANTI, Whitehall II and ALSWH (included in the AHEI-2010 score), olive oil was not measured in ALSWH and Whitehall II (included in the MDS), and EPA and DHA were not measured in InCHIANTI and ALSWH (included in the AHEI-2010). Correlations between the three dietary patterns varied considerably, see online Supplementary file 1, Table S3.

**Table 2.** Overview of dietary pattern scores, per cohort presented is the median (IQR)

	InCHIANTI	LASA	Whitehall II	NESDA	HELIUS	ALSWH
MDS (range 0–55)	29.0 (4)	33.0 (6.5)	32.0 (7.0)	31.0 (6.0)	31.0 (8.0)	26.0 (5.0)
AHEI-2010 (range 0–110)	38 (12)	58.0 (13.0)	45.0 (12.0)	59.0 (15.6)	57.0 (14.5)	46.3 (13.6)
DASH (range 8–40)	24 (6)	22.0 (6.0)	21.0 (6.0)	21.0 (6.0)	21.0 (6.0)	24.0 (6.0)

ALSWH – FFQ did not estimate olive oil, trans-fat and EPA + DHA. Whitehall II – FFQ did not estimate olive oil or trans-fat.

InCHIANTI – FFQ did not estimate trans-fat. LASA, HELIUS and NESDA – FFQ did not estimate sodium/salt.

### Cross-sectional meta-analysis

**Fig. 1.**
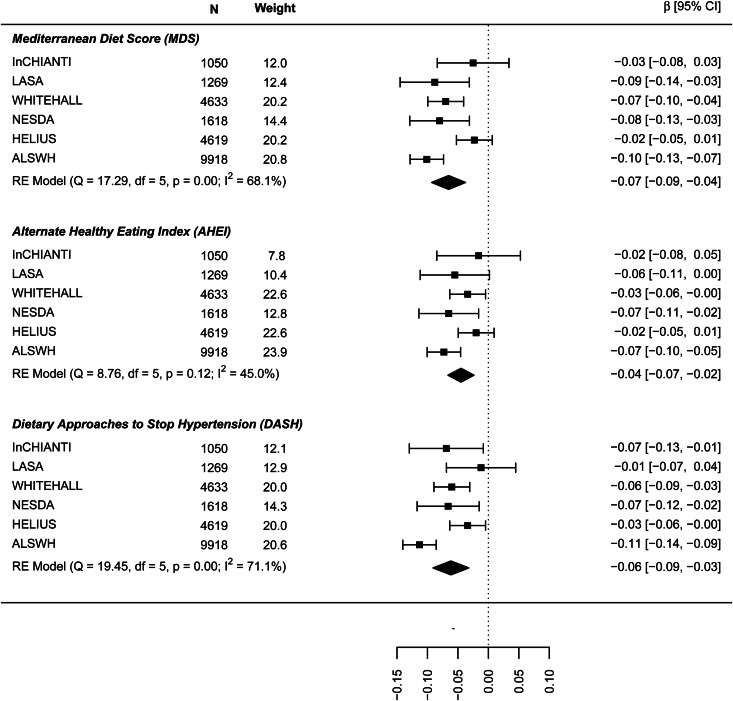
Cross-sectional association between dietary patterns and continuous ‘depressive symptoms’.

Figure 1 shows the results for the meta-analyses of the linear association between dietary patterns and depressive symptoms for the model, including all covariates and BMI. Results are presented in online Supplement 2, Table S6. All global regression coefficients reported significant estimates, the MDS score (*β* = −0.065: 95% CI −0.094 to −0.036), the DASH diet (−0.061: −0.092 to −0.030) and AHEI-2010 score (−0.045: −0.066 to −0.024). As both outcome and predictor variables were standardised estimates can be interpreted as such: a 0.04 to 0.06 standard deviation (s.d.) lower depressive symptoms score per 1 s.d. higher dietary pattern score. ALSWH might be a relevant source of heterogeneity because it reported the highest effect sizes with the lowest variances. However, the exclusion of ALSWH had little impact on the overall estimates (data not shown).

**Fig. 2.**
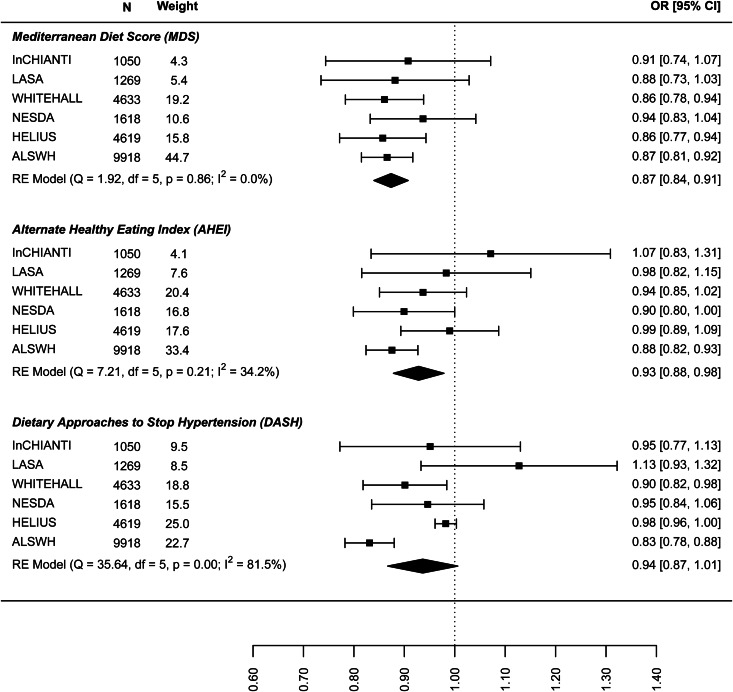
Cross-sectional association between dietary patterns and ‘high depressive symptoms’.

**Fig. 3.**
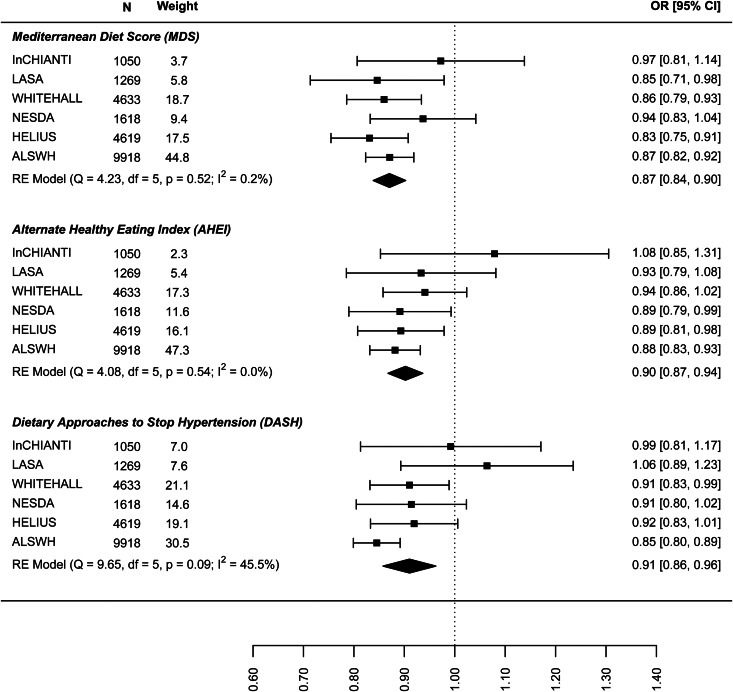
Cross-sectional association between dietary patterns and ‘high depressive symptoms e/o medications’.

The results on the association between dietary patterns and high depressive symptoms (dichotomous) are reported in Fig. 2 (see online Supplement 2, Table S6). 1 s.d. higher dietary pattern score was associated with an approximately 10% lower odds of high depressive symptoms. The MDS score seems to have a stronger association with high depressive symptoms (OR 0.874, 95% CI 0.840–0.908) than AHEI-2010 (OR 0.939: 0.861–1.018) and DASH diet (OR 0.939: 0.861–1.018). Similar results are shown in Fig. 3 for the association between dietary patterns and ‘high depressive symptoms e/o meds’. Lower values of *I*^2^ characterise the logistic analyses. In particular, *I*^2^ is zero for MDS score and AHEI-2010 score, while it is 86.0% (high depression) and 48.2% (high depression e/o med) for the DASH diet. In the analyses with dichotomous outcomes, it is the ALSWH study that is the main source of heterogeneity. In sensitivity analyses where we adjusted for WC instead of BMI and in which the ALSWH study was excluded due to lack of measures of WC, heterogeneity (*I*^2^) was 0 for all three dietary patterns scores whereas the estimates in the models correcting for BMI did not differ from the models correcting for WC. For example correction for WC in the association between the MDS and ‘high depressive symptoms e/o meds’ resulted in an OR of 0.86, while correction for BMI resulted in an OR of 0.87 (online Supplementary file 2, Table S6).

### Prospective analysis

**Fig. 4.**
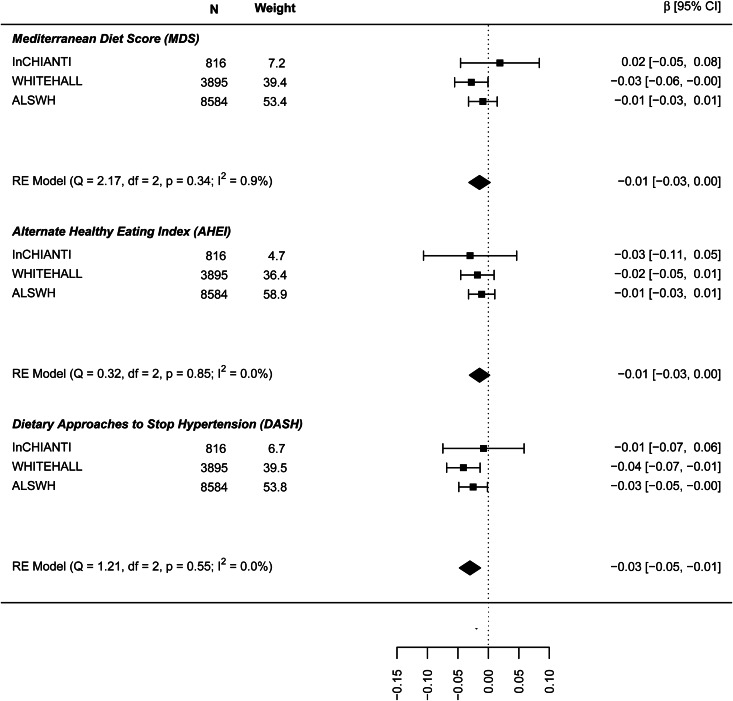
Prospective association between dietary patterns at baseline and ‘depressive symptoms’ at follow-up.

**Fig. 5.**
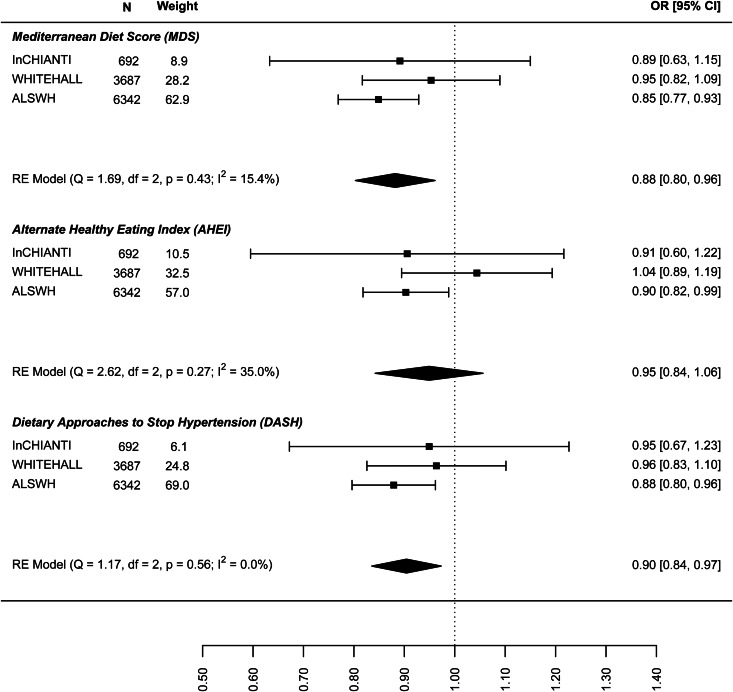
Prospective association between dietary patterns at baseline and incidence of ‘high depressive symptoms’ at follow-up.

**Fig. 6.**
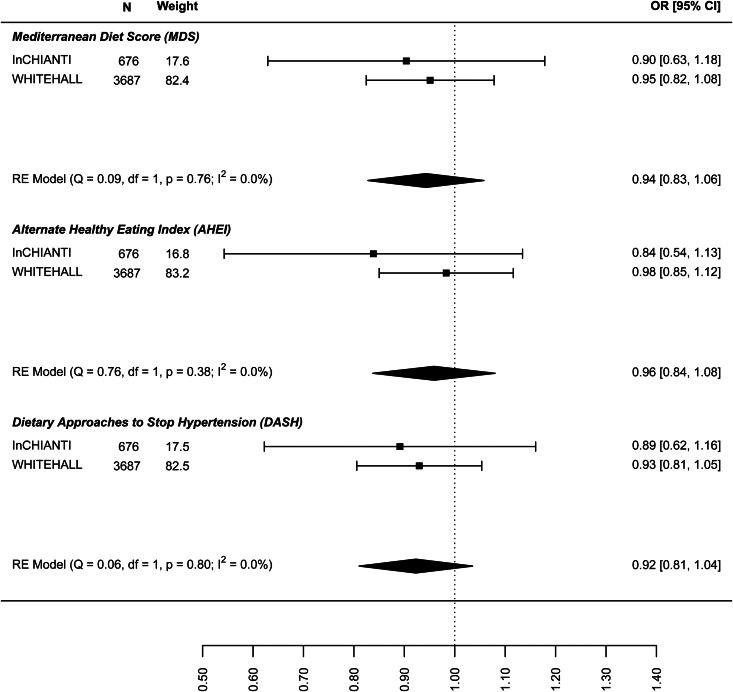
Prospective association between dietary patterns at baseline and incidence of ‘high depressive symptoms e/o medications’ at follow-up.

In the analysis of the association between baseline dietary pattern and subsequent change in continuous depressive symptoms at follow-up (Fig. 4) the estimates were only significant for the DASH diet (−0.030: −0.047 to −0.013) meaning a 0.03 decrease in standard deviation (s.d.) of depressive symptoms score per 1 s.d. increase in DASH diet score. Figure 5 presents the results for the association between dietary patterns at baseline and subsequent incidence of high depressive symptoms (5–6 years later). In this case the ORs are significant and similar to the cross-sectional analysis: the MDS (0.876: 0.810–0.943), AHEI-2010 (0.946: 0.851–1.040), and DASH (0.904, 0.836–0.973). Finally, Fig. 6 represents the association between dietary patterns and baseline and subsequent incidence of high depressive symptoms e/o meds. This analysis does not include the ALSWH cohort as medication use was not measured at follow up in this cohort. The ORs are similar to the analysis presented in Fig. 5 but as might be expected, the confidence intervals are wide and the estimate is not statistically significant. The tests *Q* for heterogeneity were not significant and the *I*^2^ present zero or negligible values for both continuous and dichotomous longitudinal outcomes. See online Supplement 2, Table S6.

## Discussion

This harmonised meta-analysis aimed to assess the association between dietary patterns and depressive symptoms and included data of over 23 000 persons from six cohorts representing varied populations in terms of age, sex, ethnicity and risk/vulnerability of depression. We observed an inverse association between three different *a priori* defined dietary patterns and depressive symptoms in cross-sectional as well as prospective analyses over a time frame of 5–6 years.

A recent meta-analysis observed that healthy dietary patterns, operationalised using *a priori* and *a posteriori* approaches, were prospectively associated with lower depressive symptoms (Molendijk *et al*., 2018). However, in that study heterogeneity was high, with *I*^2^ being 88.3 for healthy dietary patterns in general and 66.0 for Mediterranean dietary patterns. Another recent meta-analysis of *a priori* defined dietary patterns that included similar dietary patterns as the current study found an inverse association between healthy dietary patterns and depressive symptoms, with most consistent findings for the Mediterranean dietary pattern. Although heterogeneity in this study was lower in some of the meta-analyses (*I*^2^ for the Mediterranean dietary pattern was 34.4), the authors stated that differences between studies in the assessment of covariates limited the comparability between studies (Lassale *et al*., 2018). In the current study, we aimed to minimise heterogeneity by standardising variables at the individual cohort level as well as the inclusion of a wide array of potential confounders/covariates in the analyses. Heterogeneity was lower in cross-sectional analysis where we included the use of anti-depressive medication in defining high depressive symptoms implying that differences in depressive symptom measures between studies may account for the differences observed. Removal of individual studies from meta-analyses revealed that no single study was consistently responsible for heterogeneity.

A recent review observed that adherence to healthy dietary patterns was not associated with depression in studies that controlled for depressive symptoms at baseline (Molendijk *et al*., 2018). We corrected for baseline depressive symptoms in prospective analyses of the continuous outcome measure and excluded participants with ‘high depressive symptoms’ at baseline in the analysis of the dichotomous outcome at follow-up. In both approaches we found an inverse association between dietary pattern scores and depressive symptoms. Our standardised approach may explain the difference with previous findings (Molendijk *et al*., 2018).

Associations between the three dietary patterns in this study and depressive symptoms were consistent implying that all ‘healthy’ dietary patterns may contribute to the prevention of depressive symptoms. Estimates were derived from standardised confounders and standardised outcomes meaning we can compare the coefficients from each dietary pattern because they are expressed on the same scale. Findings for the MDS could be considered most consistent in that the heterogeneity was zero in most of the analyses. However, differences in effect size between the dietary patterns were quite minimal. The three dietary patterns do differ in some respects: only the MDS includes fish and olive oil, the MDS scores intake of high-fat dairy negatively while the DASH scores low fat dairy positively, just to name a few of the differences (see online Supplementary file 1, Table S2). Furthermore, the scoring of the dietary patterns used our analysis differs somewhat, the MDS and AHEI-2010 assign points according to the absolute amount that is consumed whereas the DASH scores are based on relative intakes, i.e. population quintiles. This is reflected in the low correlation between the dietary pattern scores within cohorts. Nonetheless, the patterns all score recognised elements of a healthy diet positively: fruit, vegetables, whole grains, legumes; and red and processed meats negatively.

### Strengths and limitations

The main strength of this study was the use of a standardised protocol that specified the adjustment for a large number of potential confounders, including chronic diseases and body weight. Included were three commonly applied dietary pattern scores to facilitate the comparability of the findings with the existing literature on diet and depression. It is, however, possible that the included dietary patterns do not represent the most optimal mix of foods for the prevention of depression. We specified the step-wise inclusion of potential confounders to examine their potential effect. Models were corrected for total energy intake, the presence of chronic disease and BMI, factors which could also potentially mediate the association between dietary patterns and depression, so it could be argued that there is potential for overcorrection. Indeed the largest change in estimates was observed in the models before and after correction for energy intake. For example, the association between the MDS and continuous depressive symptoms in the InCHIANTI study was −0.04 (model 1) and became smaller with adjustment for lifestyle factors and energy intake: −0.03 (model 2). However, correcting for both energy intake and BMI is common practice in nutritional epidemiology as a way of reducing the influence of residual variation in the regression analysis (Willett *et al*., 1997; Freedman *et al.*, 2011). In subsequent models we observed minimal changes in the association with the addition of confounders; the change in estimate in most cases was lower than 10% (see online Supplementary file 2, Table 6 for an overview of the change in estimate with adjustment). This implies that the association between dietary patterns and depressive symptoms observed in this study is rather robust.

A limitation is that we were only able to include three prospective cohorts, although together, these studies represent more than 11 000 individuals with widely differing characteristics. Secondly, depressive symptoms measured on a continuous scale were not normally distributed with many zero values. We log-transformed the outcome variable to compensate for lack of normal distribution and also ran the analyses using Spearman's partial correlations instead of regression analysis and found similar outcomes (results not shown). The presence of zero values cannot be solved either by transformation or non-parametric correlations but is somewhat compensated by dichotomising the outcome variable. In both approaches results were in line with each other. Thirdly, we did not study the effect of obesity but included BMI as a continuous variable in the analyses. Adding BMI did not have a large effect on the regression coefficients. For example, in HELIUS the association between the MDS and ‘high depression e/o meds’ was 0.827 (s.e. 0.039) in model 3 and 0.831 (s.e. 0.039) when BMI was included (online Supplementary file 2, Table S3). In sensitivity analyses with WC instead of BMI differences in effect size were small. In HELIUS, adjusting for WC instead of BMI in the aforementioned example resulted in an OR of 0.830 (s.e. 0.039). Fourthly, the depression scales used in this study do differ from each other and as most cohorts did not have clinical diagnoses of depression we focused on symptom scores, which is a common approach in population/cohort studies. The variation in depression measures used by each cohort is the reason we chose to standardise the outcome ‘depressive symptoms’ for the linear regression, and to perform a harmonised meta-analysis instead of an individual participant meta-analysis.

Finally, given that mental health outcomes are often clustered within individuals it is conceivable that healthy dietary patterns are also beneficial for other outcomes. For example, a study within the NESDA cohort examined depressive and anxiety disorders and found the comorbidity between these disorders to be ~67%, within this cohort it was observed that the associations with food groups were generally present for both depression and anxiety patients (Gibson-smith *et al*., 2019). Unfortunately, we could only study depression outcomes in this analysis because most of the cohorts did not include other mental health conditions.

### Implications of findings

The results of this study need to be confirmed by trials conducted in the general population as it is not possible to address causality using observational data. Nonetheless, an inverse association between dietary patterns and depressive symptoms was observed in both cross-sectional and prospective studies with little influence from a range of relevant confounders. There were considerable differences between cohorts, for example, In-Chianti consisted of older adults living in a specific rural region while the ALSWH study consisted of middle-aged women sampled from across Australia and HELIUS of a multi-ethnic population in one city. Nonetheless, associations were consistent across the studies included. The association is biologically plausible based on a number of hypothesised mechanisms in which dietary components play a role (Lopresti *et al*., 2013). Finally, the findings of two recent trials that a healthy (Mediterranean) dietary pattern may be useful as an adjunct treatment for patients with depression (Jacka *et al.*, 2017; Parletta *et al.*, 2017) lends credence to the idea that a healthy diet may impact the development of depressive symptoms.

Our findings imply that public health messages to promote healthy dietary patterns high in fruit, vegetables, whole grains and low in red and processed meat could contribute to the prevention of depression in addition to other chronic diseases. Although effect sizes were small, the findings were robust implying that healthy dietary patterns could have a significant effect at the population level.

## Conclusion

Based on the findings from this harmonised meta-analysis of observational studies, we conclude that greater adherence to a healthy dietary pattern is associated with fewer depressive symptoms and a lower risk of developing depressive symptoms over time. Promotion of a healthy diet, compatible with national dietary guidelines that are developed to prevent chronic diseases, may have additional benefits for mental health.

## Supplementary material

For supplementary material accompanying this paper visit https://doi.org/10.1017/S0033291719001958. click here to view supplementary material
