# Supplementary material for: Association of a priori dietary patterns with depressive symptoms: a harmonised meta-analysis of observational studies
Source: Psychol Med. 2019 Aug 14;50(11):1872–83. doi: 10.1017/S0033291719001958 (PMC7477372; doi:10.1017/S0033291719001958)
Supplement: Supplementary file 1 [file S0033291719001958sup.zip › S0033291719001958sup001.docx]

**The role of a priori dietary patterns in depressive symptoms: a harmonized meta-analysis of observational studies**

**Supplementary file 1**

**Table 1. Overview of foods included in each of the food groups by each cohort study**

|  | InCHIANTI | Whitehall | LASA  NESDA  HELIUS | ALSWH |
| --- | --- | --- | --- | --- |
| Fruit (excl fruit juice) | All fruits | All fruits | All fruits [excluding olives and avocado] | All fruits |
| Fruit juice | Fruit and vegetable juices | Real fruit juice | Pure 100% fruit juices | Fruit juice |
| Sugar sweetened beverages | Carbonated/Soft/Isotonic Drinks, Diluted Syrups | Fizzy drinks, Squash/Cordial | 100% fruit juices (not for DASH), fruit drinks, soft drinks, syrups/lemonade, energy drinks, sport drinks | Soft drinks, cordials, sport drinks, other carbonated drinks |
| Vegetables | Leafy, fruiting and root vegetables, cabbage, onion, garlic, stalk vegetables and sprouts, mixed salad and mixed vegetables | All vegetables, including green beans, excluding peas & baked beans | All vegetables [excluding roots/tubers, plantain and potatoes] | All vegetables |
| Potatoes | Potatoes | Potatoes boiled, roasted, potato salad | All forms of potatoes (boiled, mashed, oven prepared) [excluding French fries and other fried potatoes] | Potatoes cooked without fat |
| Red and processed meat | Beef, pork, veal, mutton, lamb, horse, game, processed meat and offals | Bacon, beef, beef burgers, corned beef, ham, lamb, liver, pork, sausages | All meat and meat products (beef, pork, organ, lamb, sausages, cold cuts) | Beef, pork, veal, lamb, sausages, salami, bacon, ham |
| Poultry | Chicken, Hen, Turkey, Rabbit | Chicken or other poultry | Chicken | Chicken |
| Fish and shellfish | Fish, Crustaceans, Molluscs | Fish fingers, battered fish, oily fish, shell fish, white fish | Lean and fatty fish, shellfish | Fish |
| Legumes | All Legumes | Lentils, Peas, Baked beans, Tofu, Soy meats | Beans and lentils (including in soup), tofu and meat substitutes based on soy | Beans, lentils, tofu |
| Nuts | Nuts, Seeds (+Nut Spread) | Nuts, peanut butter | Nuts, peanuts, peanut butter | Nuts, peanut butter |
| Whole grains | Wholegrain bread.  (durum wheat pasta) | Brown/ wholemeal bread, Wholegrain cereal, wholegrain pasta, porridge, brown rice | Wholemeal/brown/multigrain bread, wholemeal/brown/multigrain bread rolls, rye bread, wholemeal crackers; oats, muesli, other wholegrain cereals; wholemeal pasta, brown rice, bulgur, couscous | High fibre/wholemeal bread, rye bread, multi-grain bread, wholegrain cereal, porridge, muesli |
| Low-fat dairy products | Skim milk, reduced-fat milk, yoghurt, ricotta or cottage cheese, low fat cheese | Skimmed/ semi skimmed milk, cottage cheese | Low fat/fat-free yogurt and quark (≤1.5g fat/100ml); reduced-fat/skim milk, chocolate milk and yogurt drink (≤1.5g fat/100ml); low-fat cheese, cottage cheese, ricotta, goat cheese | Skim milk, reduced-fat milk, yoghurt, ricotta or cottage cheese, low fat cheese |
| High-fat dairy products | Full cream milk, flavoured milk drinks, hard, firm, cream and soft cheeses | Full fat milk, yoghurt, cheese, single/double cream, ice cream | Full fat/low fat yoghurt and quark (>1.5g fat/100ml); full fat/reduced-fat milk, chocolate milk; desserts; ice cream; cream; evaporated milk, coffee creamer; high fat hard and soft cheese | Full cream milk, flavoured milk drinks, ice cream, hard, firm, cream and soft cheeses |
| Olive oil | Olive oil used for cooking, frying, in dressings and as a dip for bread | N.A. | Olive oil used for cooking, frying, in dressings and as a dip for bread (g/d) | N.A. |
| EPA + DHA | N.A. | only available +ALA, derived from all food items | Derived from all food items (g/d) | N.A. |
| Sodium | Derived from all food items | Derived from all food items | Not calculated from the FFQ | Derived from all food items |
| Alcoholic drinks | Beer, wine, liqueur, spirits and other alcohol containing beverages | Wine, beer, spirits, port wine, liqueur | Beer, wine, liqueur, gin and other alcohol containing beverages | Beer, wine, fortified wines, port, spirits, liqueurs |

**Table 2: Operationalization of Dietary pattern scores**

Components of the three included a priori dietary patterns (+ indicates that the dietary pattern scores this food group positively, - indicates a negative scoring)

| **Food component** | **Med. Diet** Panagiotakos 2006 (servings/week) **scoring based on specific intakes** | **AHEI-2010** Chuive 2012 (servings/d) **Scoring based on specific intakes or deciles** | **DASH** Fung 2008 (servings/d) **scoring based on quintiles of intake** |
| --- | --- | --- | --- |
| Fruit (excl fruit juice) | + | + | + (incl fruit juices) |
| Vegetables (excl all potatoes) | + | + | + |
| Potatoes (excluding fried potatoes) | + | n/a | n/a |
| Red meat | - Meat and meat products (excl poultry) | - Meat and meat products (excl poultry) | - Meat and meat products (excl poultry) |
| Fish and shellfish | + (included shellfish) | n/a | n/a |
| Poultry | - | n/a | n/a |
| Nuts and Legumes | + (only legumes) | + | + |
| Whole grains | + (non-refined cereals) | + | + |
| Dairy products | - (full fat) | n/a | + (low fat) |
| Sugar Sweetened beverages | n/a | - (incl fruit juice) | _ |
| Olive oil | + | n/a | n/a |
| EPA + DHA | n/a | + | n/a |
| PUFA (% energy) | n/a | + | n/a |
| Sodium | n/a | - | - |
| Alcohol – converted to ethanol | Alcoholic beverages (ml/day, 100ml=12g ethanol) | - | n/a |
| Trans fat | n/a | - (% of energy) | n/a |
| **Range of scoring** | 0-55 | 0-110 | 8-40 |

n/a - not applicable for the scoring of this dietary pattern

**What is 1 Serving?**

Not all publications have clear guidelines for what they consider a serving to be. Where this is not specified we used the following:

Dairy: 150 ml milk;

150 g yoghurt;
40 g hard cheese;

80 g fresh and soft cheese

Fruit: one serving or one piece = 100g

Vegetables: 100g

Potatoes: 60g

Cooked cereals (rice, pasta, cooked porridge): 60g
Uncooked cereals (muesli, cornflakes etc): 35g

Bread and crackers: 35g

Red meat, chicken: 100g

Fish and shellfish: 100g

Egg: 50g

Sugar Sweetened Beverages and fruit juice: 200ml

Nuts and seeds: 25g

Peanut butter: 15g

Legumes: 125g

Alcoholic beverages: beer 250ml; wine 100 ml; liqueur/spirit 40ml

**What is whole grain?**Whole grain includes products that consist of ≥25g wholemeal flour.
Includes: wholemeal bread; brown bread; brown bread with seeds/grains; wholemeal crackers; wholemeal raisin and muesli bread/bread rolls; rye bread; oats; muesli; other wholegrain cereals (Weetabix); wholemeal pasta; brown rice; bulgur; wholemeal couscous; quinoa.

**What is Low fat dairy?**Milk: half fat (1.5g fat/100ml) and skim/low fat milk

Low fat hard cheese

Cottage cheese, ricotta, fresh goats cheese,

Yoghurt <1.5g fat/100g (half fat or fat free)

Mozzarella – varies depending on the milk used. It can be counted as low fat only if it’s made from half fat/low fat milk. As we cannot ascertain this from FFQ data we assume that it is high fat.

All other dairy = High fat dairy.

**DASH DIET** – scored on the basis of Fung et al 2008
Serving sizes defined based on guidelines (above).

**MDS** – Panagiotakos 2007
Serving sizes defined based on guidelines (above).

In addition, In many of the cohorts olive oil was calculated in ml per day, thus we applied the following scoring:

| N of portions olive oil (15ml) per week | 0 | 0-0.5 | 0.5- 1.0 | 1.0-2.99 | 3-6.999 | 7 |
| --- | --- | --- | --- | --- | --- | --- |
| score | 0 | 1 | 2 | 3 | 4 | 5 |

Alcohol – 100ml is based on wine, which includes approximately 12g of ethanol. Thus use 12g ethanol as indicative of one serving instead of 100ml in order to account for differences in the alcohol content of different drinks. Thus if participant consumes 30g ethanol(alcohol) per day they are actually consuming 30/12 = 2.5 servings.

**AHEI-2010** Chuive 2012
Serving sizes specified in original publication, exceptions:

Serving size of bread is not specified – ***use 35g***
raw cereals are not specified – ***use 35g***
Nuts, legumes, and vegetable protein (e.g., tofu): One serving is 1 oz (1 oz = 28.35 g) of nuts or 1 tablespoon (15 mL) of peanut butter. legumes not specified – ***use 125g***

Table 3: Spearman’s correlations coefficients between dietary indices in the different cohorts

| **InChianti** | | |
| --- | --- | --- |
|  | MDS | AHEI-2010 |
| AHEI-2010 | 0.449 | 1 |
| DASH | 0.501 | 0.692 |
| **Lasa** | | |
|  | MDS | AHEI-2010 |
| AHEI-2010 | 0.680 | 1 |
| DASH | 0.588 | 0.667 |
| **Whitehall II** | | |
|  | MDS | AHEI-2010 |
| AHEI-2010 | 0.481 | 1 |
| DASH | 0.529 | 0.507 |
| **NESDA** | | |
|  | MDS | AHEI-2010 |
| AHEI-2010 | 0.722 | 1 |
| DASH | 0.660 | 0.732 |
| **HELIUS** | | |
|  | MDS | AHEI-2010 |
| AHEI-2010 | 0.573 | 1 |
| DASH | 0.615 | 0.700 |
| **ALSWH** | | |
|  | MDS | AHEI-2010 |
| AHEI-2010 | 0.605 | 1 |
| DASH | 0.698 | 0.713 |
