# Supplementary material for: Association of a priori dietary patterns with depressive symptoms: a harmonised meta-analysis of observational studies
Source: Psychol Med. 2019 Aug 14;50(11):1872–83. doi: 10.1017/S0033291719001958 (PMC7477372; doi:10.1017/S0033291719001958)
Supplement: Supplementary file 1 [file S0033291719001958sup.zip › S0033291719001958sup002.doc]

**The role of a priori dietary patterns in depressive symptoms: a harmonized meta-analysis of observational studies**

**Supplementary file 2**

Table 1: Cross-sectional association between dietary pattern and depressive symptoms
(linear regression)

|  | **MDS** | | **AHEI diet score** | | **DASH diet score** | |
| --- | --- | --- | --- | --- | --- | --- |
| **Model 0** (age, sex, ethnicity, education) | β | SE | β | SE | β | SE |
| InCHIANTI (N = 1,050) | -0.039 | 0.030 | -0.016 | 0.032 | -0.078 | 0.030 |
| Lasa (N = 1,269) | -0.094 | 0.028 | -0.062 | 0.028 | -0.011 | 0.028 |
| Whitehall (N = 4,633) | -0.078 | 0.015 | -0.040 | 0.015 | -0.076 | 0.015 |
| NESDA (N = 1,618) | -0.109 | 0.024 | -0.083 | 0.025 | -0.070 | 0.025 |
| HELIUS (N = 4,619) | -0.023 | 0.015 | -0.019 | 0.015 | -0.028 | 0.015 |
| ALSWH (N = 9,918) | -0.128 | 0.013 | -0.102 | 0.012 | -0.144 | 0.012 |
| **Model 1** (0 + Demographic variables) |  |  |  |  |  |  |
| InCHIANTI (N = 1,050) | -0.035 | 0.031 | -0.017 | 0.031 | -0.077 | 0.030 |
| Lasa (N = 1,269) | -0.085 | 0.028 | -0.055 | 0.028 | -0.009 | 0.028 |
| Whitehall (N = 4,633) | -0.066 | 0.014 | -0.034 | 0.015 | -0.069 | 0.015 |
| NESDA (N = 1,618) | -0.079 | 0.024 | -0.065 | 0.024 | -0.057 | 0.024 |
| HELIUS (N = 4,619) | -0.016 | 0.015 | -0.016 | 0.014 | -0.025 | 0.014 |
| ALSWH (N = 9,918) | -0.132 | 0.013 | -0.104 | 0.013 | -0.147 | 0.013 |
| **Model 2** (1 + Lifestyle and Energy intake) |  |  |  |  |  |  |
| InCHIANTI (N = 1,050) | -0.025 | 0.031 | -0.016 | 0.035 | -0.069 | 0.031 |
| Lasa (N = 1,269) | -0.094 | 0.029 | -0.058 | 0.029 | -0.015 | 0.029 |
| Whitehall (N = 4,633) | -0.070 | 0.015 | -0.036 | 0.015 | -0.060 | 0.015 |
| NESDA (N = 1,618) | -0.081 | 0.025 | -0.064 | 0.025 | -0.068 | 0.025 |
| HELIUS (N = 4,619) | -0.028 | 0.015 | -0.020 | 0.015 | -0.031 | 0.015 |
| ALSWH (N = 9,918) | -0.105 | 0.014 | -0.082 | 0.014 | -0.117 | 0.014 |
| **Model 3** (2 + chronic disease) |  |  |  |  |  |  |
| InCHIANTI (N = 1,050) | -0.025 | 0.03 | -0.016 | 0.035 | -0.069 | 0.031 |
| Lasa (N = 1,269) | -0.093 | 0.029 | -0.061 | 0.029 | -0.014 | 0.029 |
| Whitehall (N = 4,633) | -0.072 | 0.015 | -0.036 | 0.015 | -0.061 | 0.015 |
| NESDA (N = 1,618) | -0.08 | 0.024 | -0.066 | 0.024 | -0.067 | 0.025 |
| HELIUS (N = 4,619) | -0.027 | 0.015 | -0.021 | 0.015 | -0.037 | 0.015 |
| ALSWH (N = 9,918) | -0.105 | 0.014 | -0.081 | 0.014 | -0.117 | 0.014 |
| **Model 4** (3 + BMI) |  |  |  |  |  |  |
| InCHIANTI (N = 1,050) | -0.025 | 0.03 | -0.016 | 0.035 | -0.069 | 0.031 |
| Lasa (N = 1,269) | -0.088 | 0.029 | -0.055 | 0.029 | -0.012 | 0.029 |
| Whitehall (N = 4,633) | -0.07 | 0.015 | -0.034 | 0.015 | -0.06 | 0.015 |
| NESDA (N = 1,618) | -0.08 | 0.025 | -0.065 | 0.025 | -0.066 | 0.026 |
| HELIUS (N = 4,619) | -0.023 | 0.015 | -0.02 | 0.015 | -0.034 | 0.015 |
| ALSWH (N = 9,918) | -0.101 | 0.014 | -0.073 | 0.014 | -0.113 | 0.014 |
| **Model 4** (3 + WC) |  |  |  |  |  |  |
| InCHIANTI (N = 1,050) | -0.026 | 0.03 | -0.016 | 0.035 | -0.069 | 0.031 |
| Lasa (N = 1,269) | -0.087 | 0.029 | -0.053 | 0.029 | -0.009 | 0.029 |
| Whitehall (N = 4,633) | -0.071 | 0.015 | -0.034 | 0.015 | -0.059 | 0.015 |
| NESDA (N = 1,618) | -0.08 | 0.024 | -0.066 | 0.027 | -0.067 | 0.027 |
| HELIUS (N = 4,619) | -0.023 | 0.015 | -0.019 | 0.015 | -0.032 | 0.015 |
| ALSWH (N = 9,918) | N.A. | | | | | |

Table 2: Cross-sectional association between dietary pattern and high depressive symptoms
(logistic regression)

|  | **MDS** | | **AHEI diet score** | | **DASH diet score** | |
| --- | --- | --- | --- | --- | --- | --- |
| **Model 0** (age, sex, ethnicity, education) | OR | SE | OR | **SE** | OR | SE |
| InCHIANTI (N = 1,050) | 0.946 | 0.087 | 1.111 | 0.112 | 0.937 | 0.089 |
| Lasa (N = 1,269) | 0.836 | 0.071 | 0.930 | 0.076 | 1.093 | 0.090 |
| Whitehall (N = 4,633) | 0.822 | 0.036 | 0.908 | 0.041 | 0.856 | 0.039 |
| NESDA (N = 1,618) | 0.898 | 0.048 | 0.884 | 0.047 | 0.943 | 0.050 |
| HELIUS (N = 4,619) | 0.873 | 0.042 | 0.900 | 0.043 | 0.924 | 0.043 |
| ALSWH (N = 9,918) | 0.823 | 0.023 | 0.841 | 0.023 | 0.795 | 0.022 |
| **Model 1** (0 + Demographic variables) |  |  |  |  |  |  |
| InCHIANTI (N = 1,050) | 0.955 | 0.089 | 1.111 | 0.113 | 0.941 | 0.090 |
| Lasa (N = 1,269) | 0.866 | 0.070 | 0.957 | 0.079 | 1.102 | 0.093 |
| Whitehall (N = 4,633) | 0.846 | 0.037 | 0.922 | 0.041 | 0.871 | 0.040 |
| NESDA (N = 1,618) | 0.948 | 0.052 | 0.911 | 0.050 | 0.965 | 0.052 |
| HELIUS (N = 4,619) | 0.879 | 0.043 | 0.919 | 0.046 | 0.919 | 0.046 |
| ALSWH (N = 9,918) | 0.829 | 0.023 | 0.841 | 0.024 | 0.797 | 0.022 |
| **Model 2** (1 + Lifestyle and Energy intake) |  |  |  |  |  |  |
| InCHIANTI (N = 1,050) | 0.962 | 0.091 | 1.059 | 0.124 | 0.935 | 0.094 |
| Lasa (N = 1,269) | 0.864 | 0.073 | 0.968 | 0.082 | 1.120 | 0.097 |
| Whitehall (N = 4,633) | 0.856 | 0.039 | 0.930 | 0.044 | 0.897 | 0.041 |
| NESDA (N = 1,618) | 0.938 | 0.053 | 0.905 | 0.052 | 0.946 | 0.056 |
| HELIUS (N = 4,619) | 0.856 | 0.043 | 0.908 | 0.046 | 0.932 | 0.047 |
| ALSWH (N = 9,918) | 0.862 | 0.025 | 0.870 | 0.025 | 0.834 | 0.024 |
| **Model 3** (2 + chronic disease) |  |  |  |  |  |  |
| InCHIANTI (N = 1,050) | 0.908 | 0.083 | 1.071 | 0.120 | 0.952 | 0.091 |
| Lasa (N = 1,269) | 0.867 | 0.074 | 0.959 | 0.082 | 1.120 | 0.097 |
| Whitehall (N = 4,633) | 0.855 | 0.039 | 0.930 | 0.044 | 0.895 | 0.041 |
| NESDA (N = 1,618) | 0.938 | 0.053 | 0.900 | 0.051 | 0.931 | 0.054 |
| HELIUS (N = 4,619) | 0.853 | 0.044 | 0.905 | 0.047 | 0.982 | 0.011 |
| ALSWH (N = 9,918) | 0.862 | 0.025 | 0.870 | 0.025 | 0.834 | 0.024 |
| **Model 4** (3 + BMI) |  |  |  |  |  |  |
| InCHIANTI (N = 1,050) | 0.908 | 0.083 | 1.071 | 0.121 | 0.951 | 0.091 |
| Lasa (N = 1,269) | 0.882 | 0.075 | 0.983 | 0.086 | 1.127 | 0.099 |
| Whitehall (N = 4,633) | 0.861 | 0.040 | 0.937 | 0.044 | 0.901 | 0.042 |
| NESDA (N = 1,618) | 0.937 | 0.053 | 0.899 | 0.051 | 0.946 | 0.057 |
| HELIUS (N = 4,619) | 0.857 | 0.044 | 0.990 | 0.050 | 0.982 | 0.011 |
| ALSWH (N = 9,918) | 0.866 | 0.026 | 0.875 | 0.026 | 0.831 | 0.025 |
| **Model 4** (3 + WC) |  |  |  |  |  |  |
| InCHIANTI (N = 1,050) | 0.908 | 0.083 | 1.071 | 0.120 | 0.952 | 0.091 |
| Lasa (N = 1,269) | 0.882 | 0.075 | 0.985 | 0.086 | 1.135 | 0.100 |
| Whitehall (N = 4,633) | 0.860 | 0.040 | 0.936 | 0.044 | 0.903 | 0.042 |
| NESDA (N = 1,618) | 0.939 | 0.057 | 0.899 | 0.052 | 0.931 | 0.054 |
| HELIUS (N = 4,619) | 0.856 | 0.044 | 0.904 | 0.048 | 0.982 | 0.011 |

Table 3: Cross-sectional association between dietary pattern and high depressive symptoms and/or medication (logistic regression)

|  | **MDS** | | **AHEI diet score** | | **DASH diet score** | |
| --- | --- | --- | --- | --- | --- | --- |
| **Model 0** (age, sex, ethnicity, education) | OR | SE | OR | SE | OR | SE |
| InCHIANTI (N = 1,050) | 0.959 | 0.082 | 1.129 | 0.105 | 0.985 | 0.086 |
| Lasa (N = 1,269) | 0.799 | 0.060 | 0.882 | 0.067 | 1.027 | 0.078 |
| Whitehall (N = 4,633) | 0.828 | 0.035 | 0.916 | 0.039 | 0.866 | 0.037 |
| NESDA (N = 1,618) | 0.864 | 0.048 | 0.873 | 0.048 | 0.919 | 0.050 |
| HELIUS (N = 4,619) | 0.848 | 0.038 | 0.890 | 0.040 | 0.919 | 0.040 |
| ALSWH (N = 9,918) | 0.831 | 0.022 | 0.846 | 0.022 | 0.807 | 0.021 |
| **Model 1** (0 + Demographic variables) |  |  |  |  |  |  |
| InCHIANTI (N = 1,050) | 0.962 | 0.082 | 1.126 | 0.106 | 0.987 | 0.088 |
| Lasa (N = 1,269) | 0.824 | 0.063 | 0.905 | 0.070 | 1.033 | 0.080 |
| Whitehall (N = 4,633) | 0.850 | 0.036 | 0.931 | 0.040 | 0.880 | 0.038 |
| NESDA (N = 1,618) | 0.903 | 0.051 | 0.892 | 0.050 | 0.933 | 0.052 |
| HELIUS (N = 4,619) | 0.852 | 0.039 | 0.905 | 0.042 | 0.933 | 0.042 |
| ALSWH (N = 9,918) | 0.836 | 0.023 | 0.845 | 0.022 | 0.807 | 0.021 |
| **Model 2** (1 + Lifestyle and Energy intake) |  |  |  |  |  |  |
| InCHIANTI (N = 1,050) | 0.975 | 0.085 | 1.080 | 0.116 | 0.990 | 0.091 |
| Lasa (N = 1,269) | 0.832 | 0.066 | 0.920 | 0.073 | 1.057 | 0.086 |
| Whitehall (N = 4,633) | 0.858 | 0.038 | 0.935 | 0.041 | 0.906 | 0.040 |
| NESDA (N = 1,618) | 0.895 | 0.052 | 0.893 | 0.052 | 0.912 | 0.056 |
| HELIUS (N = 4,619) | 0.830 | 0.039 | 0.898 | 0.043 | 0.928 | 0.044 |
| ALSWH (N = 9,918) | 0.864 | 0.023 | 0.873 | 0.024 | 0.843 | 0.023 |
| **Model 3** (2 + chronic disease) |  |  |  |  |  |  |
| InCHIANTI (N = 1,050) | 0.971 | 0.085 | 1.078 | 0.115 | 0.991 | 0.091 |
| Lasa (N = 1,269) | 0.834 | 0.066 | 0.912 | 0.072 | 1.058 | 0.086 |
| Whitehall (N = 4,633) | 0.855 | 0.038 | 0.934 | 0.041 | 0.904 | 0.040 |
| NESDA (N = 1,618) | 0.895 | 0.052 | 0.890 | 0.052 | 0.907 | 0.053 |
| HELIUS (N = 4,619) | 0.827 | 0.039 | 0.894 | 0.044 | 0.918 | 0.044 |
| ALSWH (N = 9,918) | 0.864 | 0.023 | 0.873 | 0.024 | 0.843 | 0.023 |
| **Model 4** (3 + BMI) |  |  |  |  |  |  |
| InCHIANTI (N = 1,050) | 0.972 | 0.085 | 1.079 | 0.115 | 0.992 | 0.091 |
| Lasa (N = 1,269) | 0.846 | 0.068 | 0.933 | 0.076 | 1.064 | 0.087 |
| Whitehall (N = 4,633) | 0.860 | 0.038 | 0.941 | 0.042 | 0.910 | 0.040 |
| NESDA (N = 1,618) | 0.937 | 0.053 | 0.891 | 0.052 | 0.914 | 0.056 |
| HELIUS (N = 4,619) | 0.831 | 0.039 | 0.893 | 0.044 | 0.919 | 0.044 |
| ALSWH (N = 9,918) | 0.871 | 0.024 | 0.882 | 0.026 | 0.845 | 0.024 |
| **Model 4** (3 + WC) |  |  |  |  |  |  |
| InCHIANTI (N = 1,050) | 0.972 | 0.085 | 1.078 | 0.115 | 0.991 | 0.091 |
| Lasa (N = 1,269) | 0.850 | 0.068 | 0.938 | 0.076 | 1.074 | 0.088 |
| Whitehall (N = 4,633) | 0.861 | 0.038 | 0.942 | 0.042 | 0.913 | 0.040 |
| NESDA (N = 1,618) | 0.901 | 0.053 | 0.893 | 0.053 | 0.910 | 0.055 |
| HELIUS (N = 4,619) | 0.830 | 0.039 | 0.894 | 0.044 | 0.920 | 0.044 |
| ALSWH (N = 9,918) | N.A. | | | | | |

Table 4: Prospective association between dietary pattern and depressive symptoms
(linear regression)

|  | **MDS** | | **AHEI diet score** | | **DASH diet score** | |
| --- | --- | --- | --- | --- | --- | --- |
| **Model 0** (age, sex, ethnicity, education) | β | SE | β | SE | β | SE |
| InCHIANTI (N = 816) | 0.008 | 0.032 | -0.040 | 0.034 | -0.018 | 0.033 |
| Whitehall (N = 3,895) | -0.028 | 0.013 | -0.019 | 0.013 | -0.046 | 0.013 |
| ALSWH (N = 8,584) | -0.070 | 0.010 | -0.051 | 0.011 | -0.078 | 0.010 |
| **Model 1** (0 + Demographic variables) |  |  |  |  |  |  |
| InCHIANTI (N = 816) | 0.007 | 0.032 | -0.041 | 0.035 | -0.019 | 0.033 |
| Whitehall (N = 3,895) | -0.025 | 0.013 | -0.018 | 0.013 | -0.044 | 0.013 |
| ALSWH (N = 8,584) | -0.072 | 0.011 | -0.052 | 0.011 | -0.079 | 0.011 |
| **Model 2** (1 + Lifestyle and Energy intake) |  |  |  |  |  |  |
| InCHIANTI (N = 816) | 0.018 | 0.032 | -0.031 | 0.039 | -0.009 | 0.034 |
| Whitehall (N = 3,895) | -0.025 | 0.014 | -0.017 | 0.013 | -0.039 | 0.014 |
| ALSWH (N = 8,584) | -0.061 | 0.012 | -0.043 | 0.011 | -0.068 | 0.011 |
| **Model 3** (2 + chronic disease) |  |  |  |  |  |  |
| InCHIANTI (N = 816) | 0.020 | 0.032 | -0.029 | 0.039 | -0.008 | 0.034 |
| Whitehall (N = 3,895) | -0.026 | 0.014 | -0.017 | 0.013 | -0.04 | 0.014 |
| ALSWH (N = 8,584) | -0.014 | 0.012 | -0.018 | 0.011 | -0.027 | 0.011 |
| **Model 4** (3 + BMI) |  |  |  |  |  |  |
| InCHIANTI (N = 816) | 0.019 | 0.033 | -0.03 | 0.039 | -0.008 | 0.034 |
| Whitehall (N = 3,895) | -0.028 | 0.014 | -0.018 | 0.014 | -0.041 | 0.014 |
| ALSWH (N = 8,584) | -0.009 | 0.012 | -0.011 | 0.011 | -0.025 | 0.012 |
| **Model 4** (3 + WC) |  |  |  |  |  |  |
| InCHIANTI (N = 816) | 0.022 | 0.033 | -0.028 | 0.039 | -0.008 | 0.034 |
| Whitehall (N = 3,895) | -0.027 | 0.014 | -0.017 | 0.014 | -0.041 | 0.014 |
| ALSWH (N = 8,584) | N.A. | | | | | |

Table 5: Prospective association between dietary pattern and high depressive symptoms
(logistic regression)

|  | **MDS** | | **AHEI diet score** | | **DASH diet score** | |
| --- | --- | --- | --- | --- | --- | --- |
| **Model 0** (age, sex, ethnicity, education) | OR | SE | OR | SE | OR | SE |
| InCHIANTI (N = 692) | 0.968 | 0.130 | 1.077 | 0.152 | 0.971 | 0.134 |
| Whitehall (N = 3,687) | 0.952 | 0.067 | 1.037 | 0.074 | 0.955 | 0.068 |
| ALSWH (N = 6,342) | 0.834 | 0.038 | 0.901 | 0.040 | 0.869 | 0.039 |
| **Model 1** (0 + Demographic variables) |  |  |  |  |  |  |
| InCHIANTI (N = 692) | 0.934 | 0.128 | 0.958 | 0.140 | 0.995 | 0.138 |
| Whitehall (N = 3,687) | 0.956 | 0.068 | 1.037 | 0.074 | 0.954 | 0.068 |
| ALSWH (N = 6,342) | 0.831 | 0.038 | 0.897 | 0.039 | 0.867 | 0.039 |
| **Model 2** (1 + Lifestyle and Energy intake) |  |  |  |  |  |  |
| InCHIANTI (N = 692) | 0.939 | 0.132 | 0.964 | 0.162 | 0.987 | 0.144 |
| Whitehall (N = 3,687) | 0.961 | 0.070 | 1.040 | 0.076 | 0.969 | 0.070 |
| ALSWH (N = 6,342) | 0.850 | 0.040 | 0.909 | 0.043 | 0.890 | 0.041 |
| **Model 3** (2 + chronic disease) |  |  |  |  |  |  |
| InCHIANTI (N = 692) | 0.943 | 0.136 | 0.952 | 0.162 | 0.973 | 0.143 |
| Whitehall (N = 3,687) | 0.960 | 0.070 | 1.047 | 0.076 | 0.969 | 0.071 |
| ALSWH (N = 6,342) | 0.849 | 0.040 | 0.908 | 0.043 | 0.890 | 0.042 |
| **Model 4** (3 + BMI) |  |  |  |  |  |  |
| InCHIANTI (N = 692) | 0.891 | 0.132 | 0.906 | 0.159 | 0.949 | 0.141 |
| Whitehall (N = 3,687) | 0.953 | 0.070 | 1.044 | 0.076 | 0.964 | 0.070 |
| ALSWH (N = 6,342) | 0.849 | 0.041 | 0.903 | 0.043 | 0.879 | 0.042 |
| **Model 4** (3 + WC) |  |  |  |  |  |  |
| InCHIANTI (N = 692) | 0.916 | 0.134 | 0.942 | 0.162 | 0.970 | 0.144 |
| Whitehall (N = 3,687) | 0.962 | 0.070 | 1.050 | 0.077 | 0.971 | 0.071 |
| ALSWH (N = 6,342) | N.A. | | | | | |

Table 6: Meta-analysis results

|  | **Model** | **Score** | **β** | **SEβ** | **95% C.I.** | **p** | **Q** | **Q.p** | **I2** | **H2** | **β variation (%)** |
| --- | --- | --- | --- | --- | --- | --- | --- | --- | --- | --- | --- |
| **Cross-sectional association between dietary pattern and depressive symptoms (linear regression)** | **Model 3** | MDS | -0.068 | 0.014 | (-0.096;-0.040) | <0.001 | 17.534 | 0.004 | 68.673 | 3.192 | Ref |
|  | **+ Chronic disease** | AHEI | -0.048 | 0.012 | (-0.071;-0.025) | <0.001 | 11.022 | 0.051 | 53.489 | 2.150 | Ref |
|  |  | DASH | -0.063 | 0.015 | (-0.093;-0.034) | <0.001 | 20.194 | 0.001 | 71.983 | 3.569 | Ref |
|  | **Model 3** | MDS | -0.065 | 0.014 | (-0.093;-0.038) | <0.001 | 17.292 | 0.004 | 68.104 | 3.135 | -4.4 |
|  | **+ chronic disease** | AHEI | -0.045 | 0.011 | (-0.066;-0.024) | <0.001 | 8.760 | 0.119 | 45.013 | 1.819 | -6.3 |
|  | **+ BMI** | DASH | -0.061 | 0.015 | (-0.091;-0.032) | <0.001 | 19.453 | 0.002 | 71.083 | 3.458 | -3.2 |
|  | **Model 3** | MDS | -0.056 | 0.014 | (-0.083;-0.029) | <0.001 | 8.889 | 0.064 | 53.662 | 2.158 | -17.7 |
|  | **+ chronic disease** | AHEI | -0.033 | 0.009 | (-0.050;-0.015) | <0.001 | 3.080 | 0.544 | 0.000 | 1.000 | -31.3 |
|  | **+ WC*** | DASH | -0.046 | 0.010 | (-0.065;-0.027) | <0.001 | 4.404 | 0.354 | 10.026 | 1.111 | -27. 0 |
|  | **Model** | **Score** | **β** | **SEβ** | **95% C.I.** | **p** | **Q** | **Q.p** | **I2** | **H2** | **β variation (%)** |
| **Cross-sectional association between dietary pattern and high depressive symptoms (logistic regression)** | **Model 3** | MDS | 0.869 | 0.017 | (0.836;0.902) | <0.001 | 2.310 | 0.805 | 0.000 | 1.000 | Ref |
|  | **+ Chronic disease** | AHEI | 0.899 | 0.019 | (0.862;0.935) | <0.001 | 4.390 | 0.495 | 3.384 | 1.035 | Ref |
|  |  | DASH | 0.933 | 0.035 | (0.865;1.000) | <0.001 | 36.714 | 0.000 | 81.446 | 5.390 | Ref |
|  | **Model 3** | MDS | 0.874 | 0.017 | (0.840;0.908) | <0.001 | 1.922 | 0.860 | 0.000 | 1.000 | -0.6 |
|  | **+ chronic disease** | AHEI | 0.929 | 0.026 | (0.878;0.979) | <0.001 | 7.211 | 0.205 | 34.159 | 1.519 | -3.3 |
|  | **+ BMI** | DASH | 0.937 | 0.035 | (0.867;1.006) | <0.001 | 35.643 | 0.000 | 81.466 | 5.396 | -0.4 |
|  | **Model 3** | MDS | 0.878 | 0.024 | (0.832;0.925) | <0.001 | 1.747 | 0.782 | 0.000 | 1.000 | -1.0 |
|  | **+ chronic disease** | AHEI | 0.928 | 0.026 | (0.878;0.979) | <0.001 | 2.479 | 0.648 | 0.000 | 1.000 | -3.2 |
|  | **+ WC*** | DASH | 0.965 | 0.023 | (0.919;1.010) | <0.001 | 6.564 | 0.161 | 30.797 | 1.445 | -3.4 |
|  | **Model** | **Score** | **β** | **SEβ** | **95% C.I.** | **p** | **Q** | **Q.p** | **I2** | **H2** | **β variation (%)** |
| **Cross-sectional association between dietary pattern and high depressive symptoms e/o medication(logistic regression)** | **Model 3** | MDS | 0.861 | 0.016 | (0.830;0.892) | <0.001 | 3.121 | 0.681 | 0.000 | 1.000 | Ref |
|  | **+ Chronic disease** | AHEI | 0.895 | 0.017 | (0.862;0.929) | <0.001 | 4.313 | 0.505 | 0.059 | 1.001 | Ref |
|  |  | DASH | 0.906 | 0.026 | (0.854;0.957) | <0.001 | 9.764 | 0.082 | 45.901 | 1.848 | Ref |
|  | **Model 3** | MDS | 0.871 | 0.016 | (0.838;0.903) | <0.001 | 4.230 | 0.517 | 0.201 | 1.002 | -1.2 |
|  | **+ chronic disease** | AHEI | 0.902 | 0.018 | (0.868;0.937) | <0.001 | 4.081 | 0.538 | 0.001 | 1.000 | -0.8 |
|  | **+ BMI** | DASH | 0.910 | 0.027 | (0.858;0.962) | <0.001 | 9.645 | 0.086 | 45.475 | 1.834 | -0.4 |
|  | **Model 3** | MDS | 0.864 | 0.022 | (0.821;0.908) | <0.001 | 2.931 | 0.569 | 0.000 | 1.000 | -0.4 |
|  | **+ chronic disease** | AHEI | 0.922 | 0.024 | (0.875;0.970) | <0.001 | 2.795 | 0.593 | 0.048 | 1.000 | -3.0 |
|  | **+ WC*** | DASH | 0.932 | 0.024 | (0.885;0.980) | <0.001 | 3.457 | 0.484 | 0.053 | 1.001 | -2.9 |
|  | **Model** | **Score** | **β** | **SEβ** | **95% C.I.** | **p** | **Q** | **Q.p** | **I2** | **H2** | **β variation (%)** |
| **Prospective association between dietary pattern and depressive symptoms (linear regression)** | **Model 3** | MDS | -0.016 | 0.009 | (-0.034;0.001) | 0.065 | 1.803 | 0.406 | 1.603 | 1.016 | Ref |
|  | **+ Chronic disease** | AHEI | -0.018 | 0.008 | (-0.034;-0.002) | 0.028 | 0.085 | 0.958 | 0.000 | 1.000 | Ref |
|  |  | DASH | -0.031 | 0.008 | (-0.047;-0.014) | <0.001 | 1.000 | 0.607 | 0.000 | 1.000 | Ref |
|  | **Model 3** | MDS | -0.014 | 0.009 | (-0.032;0.003) | 0.125 | 2.170 | 0.338 | 0.941 | 1.010 | -12.5 |
|  | **+ chronic disease** | AHEI | -0.014 | 0.008 | (-0.031;0.002) | 0.087 | 0.322 | 0.851 | 0.000 | 1.000 | -22.2 |
|  | **+ BMI** | DASH | -0.030 | 0.009 | (-0.047;-0.013) | 0.001 | 1.209 | 0.546 | 0.000 | 1.000 | -3.2 |
|  | **Model 3** | MDS | -0.012 | 0.023 | (-0.056;0.033) | 0.61 | 1.868 | 0.172 | 46.481 | 1.868 | -25.0 |
|  | **+ chronic disease** | AHEI | -0.018 | 0.013 | (-0.044;0.008) | 0.166 | 0.070 | 0.791 | 0.000 | 1.000 | 0.0 |
|  | **+ WC** | DASH | -0.036 | 0.013 | (-0.062;-0.011) | 0.005 | 0.805 | 0.369 | 0.000 | 1.000 | 16.1 |
|  | **Model** | **Score** | **β** | **SEβ** | **95% C.I.** | **p** | **Q** | **Q.p** | **I2** | **H2** | **β variation (%)** |
| **Prospective association between dietary pattern and high depressive (logistic regression)** | **Model 3** | MDS | 0.892 | 0.045 | (0.803;0.981) | <0.001 | 2.125 | 0.346 | 26.529 | 1.361 | Ref |
|  | **+ Chronic disease** | AHEI | 0.958 | 0.054 | (0.851;1.064) | <0.001 | 2.511 | 0.285 | 34.718 | 1.532 | Ref |
|  |  | DASH | 0.914 | 0.035 | (0.846;0.982) | <0.001 | 1.128 | 0.569 | 0.000 | 1.000 | Ref |
|  | **Model 3** | MDS | 0.882 | 0.040 | (0.803;0.961) | <0.001 | 1.690 | 0.430 | 15.417 | 1.182 | 1.1 |
|  | **+ chronic disease** | AHEI | 0.949 | 0.055 | (0.842;1.056) | <0.001 | 2.621 | 0.270 | 35.003 | 1.539 | 0.9 |
|  | **+ BMI (not WC)** | DASH | 0.904 | 0.035 | (0.836;0.973) | <0.001 | 1.174 | 0.556 | 0.000 | 1.000 | 1.1 |
|  | **Model 3** | MDS | 0.952 | 0.062 | (0.830;1.074) | <0.001 | 0.093 | 0.761 | 0.000 | 1.000 | -6.7 |
|  | **+ chronic disease** | AHEI | 1.030 | 0.069 | (0.895;1.166) | <0.001 | 0.366 | 0.545 | 0.000 | 1.000 | -7.5 |
|  | **+ Waist circumference*** | DASH | 0.971 | 0.064 | (0.847;1.096) | <0.001 | 0.000 | 0.995 | 0.000 | 1.000 | -6.2 |

*ALSWH not included in this analysis due to lack of data on Waist circumference.
